# Impact of Prior Fatigue on Velocity Loss as a Set-Termination Criterion in the Bench Press Exercise

**DOI:** 10.5114/jhk/214212

**Published:** 2026-04-02

**Authors:** Luis Rodiles-Guerrero, Beatriz Bachero-Mena, Moreno Miguel Sánchez-

**Affiliations:** 1 Department of Human Movement and Sport Performance, University of Seville, Seville, Spain.; 2 Science-Based Training, Physical Performance & Sports Research Center (CIRFD), Universidad Pablo de Olavide, Seville, Spain.; 3 Department of Physical Education and Sports, University of Seville, Seville, Spain.

**Keywords:** velocity-based training, volume prescription, level of effort, resistance training

## Abstract

This study analyzed the relationship between the percentage of completed repetitions with respect to the maximum that can be performed (%Rep) and the velocity loss (VL) in the bench press exercise after a previous effort (PE). Fourteen men performed four protocols (one week apart) consisting of a set to failure with 60% of one-repetition maximum (1RM) preceded by a PE with the same load but different VL magnitude (0%: PE_0_, 20%: PE_20_, 40%: PE_40_, and 60% of VL: PE_60_). Velocity against 60% 1RM (V60-load) and blood lactate concentration were measured after each PE. The relationship %Rep-VL was obtained through the coefficient of determination (R^2^) and the standard error of estimate (SEE). Absolute reliability and differences during the set to failure were calculated. V60-load decreased significantly in all protocols (except PE_0_) (p ≤ 0.05). Blood lactate concentration increased with the magnitude of effort (p ≤ 0.05). Regarding the relationship %Rep-%VL, as the %VL increased during the PE, the R^2^ decreased and the SEE increased. %Rep showed “satisfactory” absolute reliability above 15%VL, with the absolute differences being high-moderate (<10%) for all VL magnitudes (except PE_0-40_ with 60%VL). VL is a reliable and accurate set termination criterion with a PE of any magnitude, but higher reliability is observed with low-moderate levels of fatigue.

## Introduction

Muscular strength plays a crucial role across diverse populations, including recreational and elite athletes (Kraemer and Ratamess, 2004), older adults of varying functional status (Cadore et al., 2014), and pediatric populations (Hass et al., 2001). To enhance muscular strength efficiently, a well-structured resistance training (RT) program is necessary. In this context, coaches need to program each training session being aware that, depending on the configuration of the acute training variables (volume, intensity, exercise selection and order, duration, frequency, and rest intervals), the effects can change and, therefore, the physiological responses to RT (Ratamess et al., 2009; Toigo and Boutellier, 2006).

Among these acute training variables, training volume is critical for chronic muscular changes, including muscle size and force (Bird et al., 2005). Training volume denotes the total work performed within a set, a session, a week or a training program, typically calculated as sets × repetitions (Bird et al., 2005). Establishing a RT program requires matching a specific number of repetitions with each set (Bird et al., 2005). Traditionally, this has been carried out through different ways. On the one hand, one can establish a maximum number of repetitions (MNR), since there is an approximate number of repetitions per set that can be completed with each relative load (%1RM) (Nuzzo et al., 2024; Sheppard and Triplett, 2016). Nevertheless, establishing a MNR in each set requires training to failure in each session, which does not seem to be the best option when higher gains in strength and muscle size are desired (Grgic et al., 2021). Moreover, this can induce excessive mechanic and metabolic fatigue, which consequently would extend the required recovery period (Moran-Navarro et al., 2017; Pareja-Blanco et al., 2020). On the other hand, a possible solution could be not to perform the MNR in each set but a fixed number of repetitions. However, because of the high variability observed between subjects in how many repetitions they can complete using the same %1RM (Richens and Cleather, 2014; Sakamoto and Sinclair, 2006), when two individuals carry out an equal number of repetitions, the stimulus and the subsequent adaptations may differ because the real level of effort could be different.

In order to overcome the aforementioned limitations related to the training volume, velocity loss (VL) has been suggested as a critical variable to determine set termination (Gonzalez-Badillo et al., 2017). This approach is justified by the strong association observed between VL and both mechanical (r ≥ 0.91) and metabolic fatigue in the bench press (BP) and squat exercises. Specifically, VL and lactate concentrations showed R^2^ values of 0.95 and 0.97, and VL and ammonia concentrations R^2^ values of 0.89 and 0.85, for the BP and the squat, respectively (Sánchez-Medina and González-Badillo, 2011). Additionally, there is a high relationship between VL and the percentage of completed repetitions (%Rep) in relation to the MNR in the BP (R^2^ ≥ 0.96, SEE = 4.69–5.75%) (Gonzalez-Badillo et al., 2017) and the squat (R^2^ ≥ 0.96, SEE = 4.69–5.75%) (Rodriguez-Rosell et al., 2019) in men, and also in women in the BP (R^2^ ≥ 0.85, SEE = 6.85–9.81%) (Bachero-Mena et al., 2024). In this regard, the determination of %Rep based on VL within a set is possible. For instance, in the BP, a VL of approximately 60% indicates that around 80% of the total repetitions before failure have been completed. Conversely, a VL of about 20% suggests that less than the half of maximal repetitions (~40%) have been performed before failure (Rodriguez-Rosell et al., 2019). Consequently, instead of performing a fixed number of repetitions, the set could be terminated when a certain degree of muscular fatigue is detected, thereby equalizing effort (%Rep) between subjects through the VL achieved during the set, rather than a predetermined repetition count.

While the relationship between the percentage of VL (%VL) and %Rep in the BP exercise has been analyzed in isolated sets, RT sessions typically involve multiple sets. Research indicates that multiple sets yield greater strength gains compared to a single set (Peterson et al., 2004; Ralston et al., 2017). It is worth highlighting that when multiple sets are performed, the level of effort reached in the previous set can affect performance in subsequent sets (Sánchez-Moreno et al., 2023). In this regard, it has been shown that reaching a high level of effort within a set (i.e., performing repetitions to or near to task failure) causes significant disruptions to cellular homeostasis, characterized by a decrease in PCr and ATP content, increased muscle lactate concentration (González-Badillo et al., 2016; Gorostiaga et al., 2012; Sánchez-Medina and González-Badillo, 2011), and altered motor unit recruitment patterns (De Luca, 1984). Therefore, fatigue induced by a previous effort set (PE) prevents subjects from completing the scheduled repetitions (Sánchez-Moreno et al., 2023). Thus, to design RT sessions using the VL as a tool for controlling the set termination, inter-set fatigue should be taken into account since the %Rep-%VL relationship could vary from set to set (Pelland et al., 2022).

Despite the common practice of performing multiple sets in RT sessions, no studies have examined whether VL can be used to prescribe set termination after a previous set with a different level of effort. Considering the practicality of VL for determining training volume, it is important to assess if the close %Rep-%VL relationship previously observed in the literature persists when a previous set has been performed. Therefore, this study aimed to analyze the %Rep-%VL relationship during a single set to failure preceded by a previous set with different levels of effort in the BP exercise in men. It was hypothesized that VL would serve as a reliable and precise criterion for set termination when a previous set has been performed; however, its reliability would be greater under low-to-moderate fatigue than under high prior fatigue conditions, with R^2^ values expected to be higher and SEE values lower in the low-to-moderate fatigue conditions.

## Methods

### Design and Procedures

**Figure 1:**
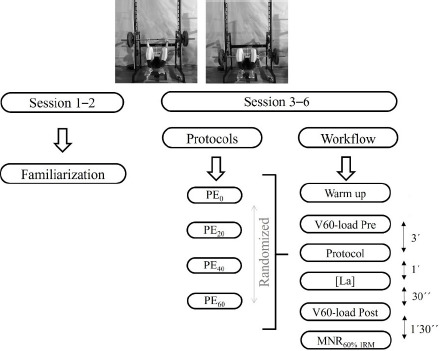
Schematic representation of the experimental design. The relative intensity of all protocols was 60% 1RM. PE_0_: previous effort set performed with a mean velocity loss of 0%; PE_20_: previous effort set performed with a mean velocity loss of 20%; PE_40_: previous effort set performed with a mean velocity loss of 40%; PE_60_: previous effort set performed with a mean velocity loss of 60%; V60-load: mean propulsive velocity attained during the 2 repetitions with the 60% of 1RM; [La]: blood lactate concentration; MNR_60% 1RM_: maximum number of repetitions completed until failure with the absolute load of 60% of 1RM at baseline measurement

A cross-over experimental methodology was employed to explore and compare how a PE with different degrees of effort during a RT session could affect the %Rep-%VL relationship during a single set to failure in the BP exercise (PE_0_, one repetition per set; 20% VL: PE_20_, ~37% of the possible repetitions per set; 40% VL: PE_40_, ~63% of the possible repetitions per set; and 60% VL: PE_60_, ~84% of the possible repetitions per set) (Gonzalez-Badillo et al., 2017). The protocols were carried out with sufficient recovery time between them (one week between each trial) (Moran-Navarro et al., 2017; Pareja-Blanco et al., 2020) and in a random sequence. To analyze and compare the mechanical and metabolic fatigue induced by each PE, performance was measured before (Pre) and after (Post) each PE using the load that elicited a velocity of approximately 0.80 m·s^−1^ (60% 1RM) at baseline (V60-load) in the BP (González-Badillo and Sánchez-Medina, 2010), along with blood lactate concentration ([La]) post the PE (Figure 1). Sessions were carried out under the supervision of the research team, scheduled at approximately the same time each day for every participant (±1 hour), and conducted in a controlled environment maintained at 20ºC and 65% humidity. Participants were advised to refrain from engaging in any other form of RT during the 48 hours preceding each protocol.

### Participants

Fourteen sports science male students (age: 24.4 ± 6.9 yrs, body mass: 76.8 ± 9.5 kg, body height: 1.77 ± 0.06 m) took part in this study. Their value of one repetition maximum (1RM) for the BP exercise was 83.0 ± 10.7 kg (1.09 ± 0.12 normalized per kg of body mass). Participants were randomly assigned to one of the four protocols (PE_0_, PE_20_, PE_40_, or PE_60_) using an ABCDDCBA counterbalancing sequence based on their 1RM at study entry, to control for potential order effects across testing sessions. All participants had a minimum of 1.5 year of RT experience, regularly included the BP exercise in their training programs, and were familiar with performing lifts at maximal intended velocity. None of the participants had musculoskeletal injuries that prevented their participation. All participants were informed of the study purpose, the procedure and the possible risks, and they all provided signed informed consent to participate in the study. The study was conducted in accordance with the ethical principles outlined in the Declaration of Helsinki and received approval from the Institutional Review Board of Virgen Macarena and Virgen del Rocío University Hospitals, Seville, Spain (protocol code: 1547-N-19; approval date: 11 October 2019).

Based on previous research (Pérez-Castilla et al., 2024; Sánchez-Moreno et al., 2024), a difference of 10 percentage points in the %Rep has been shown to have practical relevance for the RT prescription. Considering both our own sample and previous studies with similar experimental designs (González-Badillo et al., 2017; Sánchez-Moreno et al., 2024), standard deviations for %Rep typically ranged from 6.0 to 9.4. Accordingly, a 10-point difference corresponds to a moderate-to-large effect size (Cohen’s *d* ≈ 1.06–1.67; f ≈ 0.53–0.83), while a 5-point difference still represents a moderate effect (Cohen’s *d* ≈ 0.53–0.83; f ≈ 0.27–0.42). Based on these estimations, an effect size of f = 0.40 was considered appropriate for sensitivity analysis. With G*Power (version 3.1.9.7), this analysis confirmed that the current sample size (n = 14) provided 80% power to detect a minimum effect size of f = 0.33 in a one-way repeated-measures ANOVA (4 conditions), indicating that the study was adequately powered to detect practically meaningful differences in %Rep between protocols.

### Measures

#### Familiarization and Preliminary Measures

Participants completed two sessions aimed at familiarization with the testing equipment and proper technique execution in the BP exercise. Participants performed the bench press in a supine position on a bench (Bench Fitness Line, Peroga) using a Smith machine (Multipower Fitness Line, Peroga, Murcia, Spain). To standardize execution, participants placed their feet on the bench to prevent force application against the floor. Each participant initiated the movement from an upright position with elbows fully extended, then lowered the bar in a controlled and continuous manner until it made contact with the chest. To reduce any rebound effect and improve measurement consistency, a brief pause of approximately one second was implemented between the eccentric and concentric phases (Pallarés et al., 2014). Following an auditory cue from the evaluator, the concentric phase of the lift was performed at maximal voluntary velocity. An identical pause (~1 s) was applied between the concentric and subsequent eccentric phases. Participants received strong verbal encouragement throughout the task to promote maximum effort. These sessions also included assessments of body composition and the collection of personal information.

#### Resistance Exercise Protocol

During sessions 3, 4, 5 and 6, all participants performed a standardized BP warm-up protocol, which included sets of 6, 6, 4, and 3 repetitions using the following loads: the bar alone (20 kg), followed by 40% 1RM (~1.15 m∙s⁻^1^), 50% 1RM (~0.97 m∙s⁻^1^), and 60% 1RM (~0.80 m∙s⁻^1^), respectively. Each set was executed with maximal intended concentric velocity, and a 2-min rest interval was allowed separating sets. Relative loads were determined according to the general load-velocity relationship, given the well-documented strong association between %1RM and mean propulsive velocity (MPV) (González-Badillo and Sánchez-Medina, 2010). After the warm-up, participants performed 2 repetitions with the V60-load, which served as a baseline dynamic strength measure. The absolute loads (in kg) were individually tailored to ensure that the resulting MPV closely matched (± 0.03 m·s⁻^1^) the prescribed %1RM. This margin was selected based on previous research (Courel-Ibáñez et al., 2019), which demonstrated that it represents the smallest detectable change in MPV under conditions comparable to the present study (i.e., BP exercise performed on a Smith Machine with the T-Force System). Subsequently, the designated protocol was executed. All protocols had the same intensity (60% 1RM) but varied in the degree of effort (0%, 20%, 40%, and 60% VL), and were performed before a test of the MNR to failure against an absolute load equal to 60% 1RM at baseline.

#### Assessment of Mechanical Fatigue and Metabolic Stress

Mechanical fatigue was assessed by evaluating the absolute VL observed when lifting the V60-load. This metric was selected due to its ability to reflect the impact of fatigue on movement velocity from pre- to post-exercise, while also being a manageable and well-tolerated load across a wide range of individuals (Sánchez-Moreno et al., 2023). Metabolic stress was evaluated through post-exercise [La]. Approximately 5 μL of capillary blood was collected from the fingertip and assessed with a handheld lactate meter (Lactate Pro 2; ARKRAY). The device’s reliability and accuracy had been previously validated, showing coefficients of variation of 7.6%, 3.5%, and 2.7% for lactate concentrations of 1, 4, and 12 mmol·l⁻^1^, respectively (Bonaventura et al., 2015).

#### Set of the Maximum Number of Repetitions until Failure

Three minutes after the PE set, participants performed repetitions to concentric muscle failure using a load equivalent to 60% of the baseline 1RM. Task failure was defined as the inability of the activated muscles to complete another repetition within the appropriate range of motion (Jenkins et al., 2015). The execution technique adhered to the protocol outlined in the 'Familiarization and Preliminary Measures' section.

### Measurement Equipment and Data Acquisition

All repetitions were recorded at 1000 Hz using a linear velocity transducer (T-Force Dynamic Measurement System, Ergotech, Murcia, Spain). The reliability of this system had been previously established (Sánchez-Medina and González-Badillo, 2011). The velocity outcome measure used in this study was MPV, described as the segment of the concentric action during which the recorded acceleration (a) exceeded acceleration due to gravity, i.e., a ≥ –9.81 m·s⁻^2^ (Sánchez-Medina et al., 2010). Thus, MPV considered mean values only during the propulsive phase, avoiding the breaking phase that negatively influenced the computation of the mean concentric velocity with light and medium loads (up to ~ 80% 1RM) (Sánchez-Medina et al., 2010).

### Statistical Analysis

Descriptive statistics, comprising means and standard deviations (SD), were computed using conventional statistical procedures. Data normality was assessed using the Shapiro-Wilk test. The relationship between %VL during the set and %Rep completed at 60% 1RM was analyzed by fitting second-degree polynomial models. For these models, coefficients of determination (R^2^) and the standard error of estimate (SEE) were calculated. To evaluate absolute reliability, the intrasubject coefficient of variation (CV) was computed as a relative measure, using the formula: 100 × SEM / mean. Here, SEM referred to the standard error of measurement, derived from the root mean square of the intra-subject total mean square. Based on the criteria proposed by Stokes (1985), CV values ≤15% were considered indicative of satisfactory reliability. During the set to task failure after each PE, the absolute variations between the observed %Rep in the PE_20_, PE_40_, and PE_60_ protocols and the predicted %Rep in the PE_0_ protocol, for a given magnitude of VL (20, 40, and 60% of VL), were calculated using both the general and individual equations obtained from the general and individual relationship. The goal of this analysis was to evaluate the accuracy of the %Rep-%VL relationship (cross-validation) when applied across different protocols. The accuracy was classified as high (absolute error <5%), moderate (absolute error between 5% and 10%), or low (absolute error >10%) (Pérez-Castilla et al., 2024). A one-way repeated measures analysis of variance was conducted to analyze the differences between protocols in the PE set for all descriptive variables and the absolute differences in the %Rep. In addition, this analysis was used to analyze the differences between %Rep-%VL carried out after each PE set. A four (protocol: PE_0_, PE_20_, PE_40_, PE_60_) by two (time: Pre vs. Post) repeated measures ANOVA was performed to analyze the differences for the V60-load. Bonferroni post hoc tests were applied when the interaction reached significance. Bonferroni post hoc tests were used when the interaction was significant. Significance was accepted at *p* ≤ 0.05. Pre-post effect size (ES) values were calculated using Hedge’s g on the pooled SD (Hedges and Olkin, 1985). All statistical analyses were performed using SPSS software (version 20.0).

## Results

### Descriptive Characteristics of Previous Effort Set

**Figure 2:**
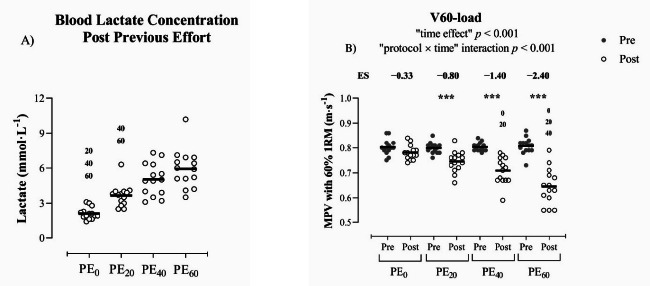
Changes in metabolic and mechanical variables. A) Blood lactate concentration; B) load that elicited 60% one-repetition maximum (~0.80 m·s^−1^) at baseline measurements (V60-load). PE_0_: previous effort set performed with a mean velocity loss of 0%; PE_20_: previous effort set performed with a mean velocity loss of 20%; PE_40_: previous effort set performed with a mean velocity loss of 40%; PE_60_: previous effort set performed with a mean velocity loss of 60%; intra-group significant differences from Pre to Post previous effort: *** p ≤ 0.001; between group significant differences (p < 0.05) with PE_0_: ^0^; PE_20_: ^20^; PE_40_: ^40^; PE_60_: ^60^

Table 1 provides the descriptive variables for the four protocols performed before the set until failure. The fastest repetition (MPV_BEST_) and the %VL did not differ from the expected target values corresponding to each protocol (Table 1). The average number of total repetitions performed during each protocol was higher as %VL increased (*p* < 0.001). Figure 2A shows the acute metabolic response to each RT protocol. In this regard, PE_60_ and PE_40_ protocols displayed significantly higher blood lactate concentration than PE_0_ (*p* < 0.001) and PE_20_ protocols (*p* < 0.001–0.05). In addition, the PE_20_ protocol exhibited significantly higher [La] than PE_0_ (*p* < 0.05) (Figure 2A).

**Table 1:** Descriptive variables for the previous effort performed with a load of 60% 1RM with different velocity loss thresholds.

PE	MPV_BEST (m∙s^−1^)_	MPV_LAST (m∙s^−1^)_	Velocity loss (%)	Rep	Load (kg)
PE_0_	0.79 ± 0.03	-	0 ± 0.0^20 40 60^	1^20 40 60^	49.0 ± 6.9
PE_20_	0.78 ± 0.04	0.62 ± 0.04	21.35 ± 2.95^40 60^	7.4 ± 1.3^40 60^	49.0 ± 7.2
PE_40_	0.79 ± 0.02	0.46 ± 0.02	42.21 ± 2.26^60^	12.7 ± 2.4^60^	48.9 ± 6.7
PE_60_	0.79 ± 0.03	0.31 ± 0.1	64.21 ± 5.45	16.7 ± 3.4	48.7 ± 6.4

Data are mean ± SD; MPV_BEST_: mean propulsive velocity of the fastest (usually first) repetition in the set; MPV_LAST_: mean propulsive velocity of the last repetition in the set; Rep: number of repetitions completed in the set. PE_0_, indicates the set performed with a mean velocity loss of 0%; PE_20_, set performed with a mean velocity loss of 20%; PE_40_, set performed with a mean velocity loss of 40%; PE_60_, set performed with a mean velocity loss of 60%; statistical significance (p < 0.001) with respect to ^20^PE20, ^40^PE40, ^60^PE60

With regard to the acute mechanical response to each RT protocol, a significant “protocol × time” and “time effect” interaction was found for the V60-load (*p* < 0.001). Significant differences (*p* < 0.001) were observed in this variable from the pre to the post PE set for all protocols, except for the PE_0_ protocol. Additionally, the PE_60_ protocol showed a significantly lower MPV at the V60-load compared to PE_0_ (*p* < 0.001) and PE_20_ protocols (*p* < 0.05). Moreover, the PE_40_ protocol induced lower MPV in this variable than the PE_0_ protocol (*p* < 0.05) (Figure 2B).

### Descriptive Characteristics of Set until Failure in the Bench Press Exercise

Table 2 summarizes the characteristics of each set until failure preceded by each PE protocol. Significant differences were found in the MPV_BEST_ values between protocols, where the PE_60_ protocol showed lower MPV_BEST_ values with respect to PE_0_ (*p* < 0.001) and PE_20_ (*p* = 0.05) protocols. Average MPVs of the last repetition and VL (%) of each set to failure were very similar after performing each protocol. Furthermore, no significant differences were observed between protocols in the number of repetitions performed and the average individual R^2^ values (Table 2).

**Table 2:** Descriptive variables for the set performed to failure against a load of 60% 1RM with a previous effort with different velocity loss thresholds.

	MPV_BEST_	MPV_LAST_	Velocity loss (%)	Rep	R^2^
PE_0_	0.77 ± 0.03^60^	0.14 ± 0.05	81.29 ± 5.86	19.5 ± 5.5	0.98 ± 0.02
PE_20_	0.75 ± 0.04^60^	0.13 ± 0.04	82.67 ± 6.13	18.8 ± 5.9	0.98 ± 0.02
PE_40_	0.74 ± 0.05	0.15 ± 0.04	79.41 ± 6.83	16.6 ± 6.1	0.97 ± 0.03
PE_60_	0.70 ± 0.05	0.15 ± 0.05	78.59 ± 7.18	14.8 ± 5.7	0.96 ± 0.06

Data are mean ± SD; 1RM: One-repetition maximum; PE_0_ indicates that the previous effort set was performed with a mean velocity loss of 0%; PE_20_, indicates that the previous effort set was performed with a mean velocity loss of 20%; PE_40_, indicates that the previous effort set was performed with a mean velocity loss of 40%; PE_60_, indicates that the previous effort set was performed with a mean velocity loss of 60%. MPV_BEST_: mean propulsive velocity of the fastest (usually first) repetition in the set; MPV_LAST_: mean propulsive velocity of the last repetition in the set; Rep: number of repetitions completed in the set; R^2^: coefficient of determination; significant differences (p ≤ 0.05) with respect to the ^60^VL60 protocol

**Figure 3:**
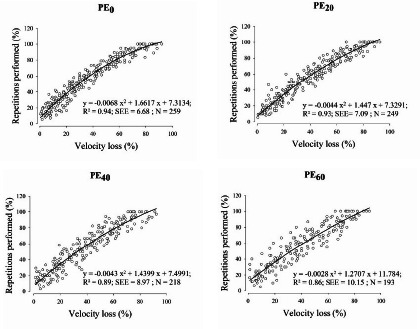
Relationships between the percentage of performed repetitions with respect to the maximum (to failure) number of repetitions that can be completed (%Rep) and the percentage of velocity loss (VL) with a load of 60% 1RM after each previous effort set (PE). *PE_0_: previous effort set performed with a mean velocity loss of 0%;* PE_20_: previous effort set performed with a mean velocity loss of 20%; PE_40_: previous effort set performed with a mean velocity loss of 40%; PE_60_: previous effort set performed with a mean velocity loss of 60%

Regarding the relationship between %Rep and %VL, it can be observed that as %VL increased in the PE set, the value of the R^2^ decreased and the SEE increased (R^2^ = 0.94, 0.93, 0.89, 0.86; SEE = 6.68%, 7.10%, 8.97%, 10.15% for PE_0_, PE_20_, PE_40_ and PE_60_, respectively) (Figure 3). No significant differences between protocols were found in the %Rep once a particular VL magnitude was attained (Table 3).

**Table 3:** Inter-subject CV and the percentage of completed repetitions out of the maximum number of repetitions to failure when a given magnitude of velocity loss is reached, with a previous effort of 0%, 20%, 40%, and 60% velocity loss.

Percentage of repetitions completed					
Velocity loss (%)	PE_0_	PE_20_	PE_40_	PE_60_	CV (%)
10	22.7 ± 5.9	21.1 ± 5.8	21.7 ± 7.7	23.2 ± 7.6	20.7
15	30.3 ± 5.8	28.0 ± 5.9	28.5 ± 7.6	29.7 ± 7.8	16.3
20	37.7 ± 5.9	34.7 ± 6.1	35.2 ± 7.6	36.1 ± 8.4	14.1
25	44.7 ± 5.9	41.2 ± 6.2	41.7 ± 7.7	42.3 ± 9.0	12.6
30	51.4 ± 5.9	47.4 ± 6.3	48.0 ± 7.9	48.3 ± 9.6	11.5
35	57.7 ± 5.8	53.5 ± 6.4	54.1 ± 8.0	54.2 ± 10.1	10.5
40	63.7 ± 5.6	59.3 ± 6.4	60.0 ± 8.0	60.0 ± 10.3	9.6
45	69.3 ± 5.3	64.8 ± 6.3	65.7 ± 7.9	65.5 ± 10.4	8.7
50	74.7 ± 5.0	70.2 ± 6.1	71.2 ± 7.7	71.0 ± 10.3	7.9
55	79.6 ± 4.6	75.3 ± 5.9	76.5 ± 7.5	76.2 ± 9.9	7.0
60	84.3 ± 4.1	80.2 ± 5.7	81.7 ± 7.2	81.4 ± 9.4	6.2
65	88.5 ± 3.8	84.9 ± 5.5	86.6 ± 7.0	86.3 ± 8.8	5.4
70	92.5 ± 3.7	89.4 ± 5.6	91.4 ± 6.8	91.2 ± 8.1	4.7
75	96.1 ± 4.0	93.6 ± 5.8	95.9 ± 7.0	95.8 ± 7.6	4.2
80	99.3 ± 4.7	97.6 ± 6.4	100.3 ± 7.4	100.3 ± 7.4	4.2

Data are mean ± SD; CV: intrasubject coefficient of variation (in bold); PE_0_ indicates that the previous effort set was performed with a mean velocity loss of 0%; PE_20_, the previous effort set was performed with a mean velocity loss of 20%; PE_40_, the previous effort set was performed with a mean velocity loss of 40%; PE_60_, the previous effort set was performed with a mean velocity loss of 60%

### Reliability and Accuracy of the Measurements

Concerning the %Rep for a given VL, it can be observed that from 20% to 80% VL, CVs varied from 14.1 to 4.2%, with higher values observed when the VL achieved in the set was lower. Nevertheless, 10% and 15% of VL showed CVs of 20.7% and 16.3%, respectively (Table 3).

**Figure 4:**
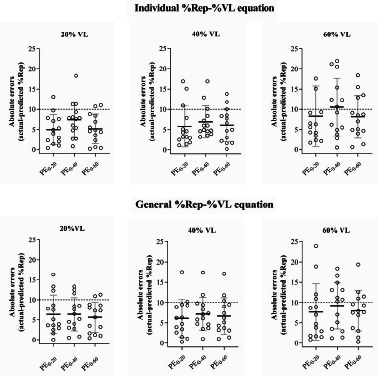
Absolute errors between the actual percentage of performed repetitions with respect to the maximum (to failure) number of repetitions that can be completed (%Rep) in the PE_20_, PE_40_, and PE_60_ protocols and the predicted %Rep in the PE_0_ protocol, when a given magnitude of velocity loss is reached (20%, 40%, and 60% of velocity loss) from the individualized (upper panels) and generalized (lower panels) %Rep-%VL equations. The dashed line represents the threshold for high accuracy (10%). PE_0_: previous effort set performed with a mean velocity loss of 0%; PE_20_: previous effort set performed with a mean velocity loss of 20%; PE_40_: previous effort set performed with a mean velocity loss of 40%; PE_60_: previous effort set performed with a mean velocity loss of 60%

Figure 4 depicts the absolute differences during the set to task failure after each PE. No significant differences in the absolute errors were noticed between protocols for any VL magnitude and regression models.

## Discussion

The present study aimed to analyze the %Rep-%VL relationship in the BP exercise when a PE with different levels of effort (0%, 20%, 40%, and 60% of VL) was performed. The findings of this study confirmed the following: i) VL in the BP exercise after a PE remained highly associated with %Rep when the level of effort in the previous set was low to moderate, with R^2^ values ranging from 0.94 to 0.89 and SEE values from 6.68 to 8.97 for PE_0_, PE_20_ and PE_40_. However, when the PE was carried out near failure (60% VL), the goodness of fit for the %Rep-%VL relationship might be compromised (R^2^ = 0.86 and SEE = 10.15); ii) when a PE had been performed, %Rep showed a satisfactory absolute reliability (intra-subject CV) for VL magnitudes > 20%; iii) moderate (absolute errors below 10%) and similar accuracy in the %Rep estimation was observed with VL magnitudes of 20, 40, and 60%; and iv) the %Rep for specific magnitudes of VL (15% to 80% VL) were similar across all protocols.

Previous studies that have analyzed VL as a set termination prescription found a high %Rep-%VL relationship in the BP exercise in an isolated set (Bachero-Mena et al., 2024; Gonzalez-Badillo et al., 2017; Rodriguez-Rosell et al., 2019; Sánchez-Moreno et al., 2021). Consistent with our results, the goodness of fit observed in the aforementioned studies using 60% 1RM (R^2^ = 0.97–0.94, SEE = ~6%) was very similar to that obtained in the present study when the set to failure was carried out during the PE_0_ protocol (i.e., PE_0_: R^2^ = 0.94, SEE = 6.68%). These similarities could derive from the fact that mechanical and metabolic fatigue induced during the PE_0_ protocol was the least that can be induced during a training set (1 repetition) (Figure 2A and 2B). Nevertheless, considering that the mechanical and metabolic stress increased proportionally with the increment of %VL accumulated during the PE (Figure 2A and 2B), the goodness of the fit decreased as a consequence (lower R^2^ and higher SEE: R^2^ = 0.94, 0.93, 0.89, 0.86; SEE = 6.68%, 7.10%, 8.97%, 10.15% for PE_0_, PE_20_, PE_40_, and PE_60_, respectively) (Figure 3). This decrease may be explained by the fact that a 3-min rest interval may be insufficient for full recovery of adenosine triphosphate and phosphocreatine levels after performing a set with a high degree of effort (~64% of VL). It should be noted that the level of effort reached during the training sets is a critical factor in the subsequent physiological adaptations associated with RT (Bird et al., 2005; Tan, 1999). In this regard, it has been shown that moderate VL thresholds (20–30% of VL) can maximize the performance and neuromuscular adaptations in the BP exercise after an 8-week velocity-based RT program with intensities between 40 to 85% 1RM (Pareja-Blanco et al., 2020; Rodiles-Guerrero et al., 2022, 2024). Therefore, athletes training with a low-to-moderate level of effort during the session can use the VL as a useful tool to determine when the set should be stopped after a PE in the BP exercise. However, when the level of effort during the session is very high (i.e., training near to muscular failure), the quality of the fit of the %Rep-VL relationship could be compromised. For this reason, the present study compared a wide range of VL thresholds (0%, 20%, 40%, and 60%) to determine how different levels of effort influence the validity of this relationship under common training conditions.

Regarding reliability after each PE, as the magnitude of VL over the set increased, the CV values for the %Rep corresponding to a given VL tended to diminish. Additionally, when VL thresholds were greater than 15%, the %Rep showed satisfactory absolute reliability (CV values lower than 15%) (Table 3). In this regard, other studies have analyzed the reliability for the BP exercise with the same load used in the present study, albeit without a PE (Gonzalez-Badillo et al., 2017; Sánchez-Moreno et al., 2021). Gonzalez-Badillo et al. (2017) reported greater absolute reliability (within-subject CV ≤ 6.6%), between two sessions (6–7 days apart), regarding the %Rep achieved at different VL (from 15% to 75%). Similarly, Sánchez-Moreno et al. (2021) reported comparable reliability values (within-subject CV = 13.7–3.6%, for VL values of 25% to 80%). Therefore, our results support that when a PE has been carried out, the %Rep at a given VL magnitude displays satisfactory absolute reliability. Nevertheless, the reduced reliability found at low VL thresholds may stem from the technical demands of using bar velocity loss to monitor volume. During the first repetitions, differences in MPV may be minimal, likely because fatigue is still low and may coexist with potentiation, introducing variability in VL. As a result, in the early stages of a set, notable differences can appear between low %VL (e.g., 0% vs. 10%) at initial %Rep values (< 30%), which increases the CV. However, as the set progresses, fatigue accumulates and causes greater drops in MPV, explaining the progressive decrease in the CV as %Rep approaches its maximum.

In the present study, the accuracy of the %Rep estimation was evaluated for each PE using cross validation. Absolute differences between the actual %Rep completed during the set to failure after reaching 20%, 40%, and 60% VL and the predicted values using the %Rep-%VL relationships were derived from the data collected during the PE_0_ protocol. It should be noted that this protocol (PE_0_) was used as baseline measurement to compare with the other protocols (PE_20_, PE_40_, and PE_60_) because there was no accumulated fatigue after this PE (just one repetition performed) (Figure 2). The accuracy was high to moderate, with absolute differences being <10% for all magnitudes of VL analyzed (except for PE_0–40_ with 60% VL) and no differences were noticed between the general and individual equations (Figure 4). It is noteworthy that even in the only case where the difference was >10% (PE_0–40_ with 60% VL), the absolute error in terms of the number of repetitions corresponded to 2.1 ± 1.4 repetitions. This means that despite the accuracy being considered low for such a case, the small difference in the number of repetitions (2.1 ± 1.4 repetitions) represents an error that can be assumed and is unlikely to have any significant impact on the acute fatigue experienced by the athlete, and consequently on long-term adaptations. Our results are in disagreement with a recent study that analyzed the accuracy of the relationship between %Rep and %VL in the squat exercise when performing multiple sets (Pérez-Castilla et al., 2024), which reported low accuracy (absolute error >10%). A possible explanation could be that in the study conducted by Pérez-Castilla et al. (2024), participants reached the task failure in each set and the recovery time (2 min) was probably not sufficient to restore the energy balance in the muscles involved (Gorostiaga et al., 2012).

Interestingly, the %Rep for specific magnitudes of VL (15% to 80% VL) were similar across PE protocols. Although non-significant (*p* > 0.05), the PE_0_ protocol showed a slightly higher %Rep for each VL magnitude (Table 3). Comparing the %Rep associated with each VL threshold to other studies that analyzed this relationship with the same intensity (60% 1RM) in the BP exercise, itis notable that our data, especially from the PE_0_ protocol, are similar (Gonzalez-Badillo et al., 2017; Sánchez-Moreno et al., 2021). Our results indicate that VL magnitude is associated with %Rep independently of the level of effort induced during a previous set.

Our results showed a close relationship between %Rep and %VL in the BP exercise when a low-to-moderate PE was induced. This allows strength and conditioning coaches to use the movement velocity (through velocity loss) to accurately determine the true degree of effort in this exercise. Therefore, practitioners have a useful tool for prescribing set termination when the goal is to maximize the performance and mechanical adaptations in the BP exercise during RT. Starting each set with low-to-moderate fatigue allows more accurate monitoring of velocity loss. Even when %Rep prediction errors exceeded 10%, the discrepancy was limited to approximately two repetitions, a difference unlikely to substantially affect acute fatigue or long-term training adaptation.

Despite the valuable findings, an important limitation of the present study should be considered when interpreting the results. All participants began each protocol at the velocity corresponding to 60% of 1RM, estimated from the general load-velocity relationship (~0.80 m·s⁻^1^). However, previous research has shown that the velocity associated with a given %1RM can vary substantially between individuals, potentially leading to mismatches in the relative load across participants (Sánchez-Moreno et al., 2021). This may explain the considerable individual variability observed in mechanical and metabolic responses in the PE. Future research should therefore examine the impact of different approaches to adjusting intensity (e.g., generalized vs. individualized equations) on the %Rep-%VL relationship during a single set to failure preceded by a prior set performed at different effort levels in the bench press exercise.

## Conclusions

In the BP exercise, the relationship between %Rep and %VL varies from set to set as fatigue increases during a PE. In our study it was observed that a previous set with a high level of effort (i.e., 60% VL) could compromise the goodness of fit between the %Rep and the VL relationship. Nevertheless, regardless of the level of fatigue induced by the previous effort (up to 15% VL), the %Rep shows satisfactory absolute reliability, and the same %VL represents a similar %Rep.
